# Dynamics of Intersexual Dominance and Adult Sex- Ratio in Wild Vervet Monkeys

**DOI:** 10.3389/fpsyg.2020.00839

**Published:** 2020-05-14

**Authors:** Charlotte Korinna Hemelrijk, Matthias Wubs, Gerrit Gort, Jennifer Botting, Erica van de Waal

**Affiliations:** ^1^Groningen Institute for Evolutionary Life Sciences, University of Groningen, Groningen, Netherlands; ^2^Department of Ecology and Evolution, University of Lausanne, Lausanne, Switzerland; ^3^Inkawu Vervet Project, Mawana Game Reserve, Kwazulu Natal, South Africa; ^4^Biometris, Wageningen University & Research, Wageningen, Netherlands

**Keywords:** the winner-loser effect, dominance hierarchy, fierceness of aggression, female dominance over males, adult sex-ratio, vervet monkeys

## Abstract

Intersexual dominance relations are important for female mammals, because of their consequences for accessing food and for the degree of sexual control females experience from males. Female mammals are usually considered to rank below males in the dominance hierarchy, because of their typical physical inferiority. Yet, in some groups or species, females are nonetheless dominant over some males (partial female dominance). Intersexual dominance, therefore, also depends on traits other than sexual dimorphism, such as social support, social exchange, group adult sex-ratio, and the widespread self-reinforcing effects of winning and losing fights, the “winner-loser effect.” The importance of sex-ratio and the winner-loser effect remains poorly understood. A theoretical model, DomWorld, predicts that in groups with a higher proportion of males, females are dominant over more males when aggression is fierce (not mild). The model is based on a small number of general processes in mammals, such as grouping, aggression, the winner-loser effect, the initially greater fighting capacity of males than females, and sex ratio. We expect its predictions to be general and suggest they be examined in a great number of species and taxa. Here, we test these predictions in four groups of wild vervet monkeys (*Chlorocebus pygerythrus*) in Mawana game reserve in Africa, using 7 years of data. We confirm that a higher proportion of males in the group is associated with greater dominance of females over males; a result that remains when combining these data with those of two other sites (Amboseli and Samara). We additionally confirm that in groups with a higher fraction of males there is a relatively higher (a) proportion of fights of males with other males, and (b) proportion of fights won by females against males from the fights of females with any adults. We reject alternative hypotheses that more dominance of females over males could be attributed to females receiving more coalitions from males, or females receiving lowered male aggression in exchange for sexual access (the docile male hypothesis). We conclude that female dominance relative to males is dynamic and that future empirical studies of inter-sexual dominance will benefit by considering the adult sex-ratio of groups.

## Introduction

In many group-living animals there is a dominance hierarchy and dominant individuals usually have priority of access to resources (Drews, 1993). In terms of dominance between the sexes, females may benefit from dominating males for several reasons, for instance by:

(A) suffering less sexual coercion (Smuts and Smuts, 1993; Muller and Wrangham, 2009; Surbeck and Hohmann, 2013; Palombit, 2014),
(B) having more freedom in choosing mates (Soltis, 1999; Muller and Wrangham, 2009; but see Rosenblum and Nadler, 1971),
(C) being able to protect their infants better against harassment by males (Smuts and Smuts, 1993; Muller and Wrangham, 2009).
(D) having more opportunity to lead group movement, which may result in feeding priority (Waeber and Hemelrijk, 2003; Overdorff et al., 2005; Van Belle et al., 2013).

However, only in rare cases females are dominant over males. This happens in primates in lemurs where the sexes have approximately equal body size and in spotted hyenas where females are slightly larger than males. In most mammals, however, males are larger and more dangerous in their weaponry (e.g., they have larger canines) than females (Clutton-Brock, 2016) and therefore, are usually considered to be dominant over females. If body size and weaponry (so-called prior attributes) alone contributed to an individual’s position in the hierarchy, then in those species where each adult male is larger than each adult female in the group (as in many mammalian species), all males should always be dominant over all females (the so-called prior attribute hypothesis, Chase et al., 2002). However, this is not always the case and smaller females are sometimes observed to beat larger males, so-called partial female dominance (Smuts, 1987). This is usually explained as a consequence of coalitions among females against males (Smuts, 1987; Smuts and Smuts, 1993; Parish, 1994; Setchell et al., 2006; White and Wood, 2007), but could also be a consequence of males reducing aggression to females for getting sexual access to them, referred to as the docile male hypothesis (Surbeck and Hohmann, 2013). Most interestingly for the present paper, a computational model, called DomWorld, showed that competitive interactions may make females dominant over some males through the self-reinforcing effects of winning and losing fights; the winner-loser effect (Hemelrijk et al., 2008). These self-reinforcing effects imply that, after losing a fight, the loser is more likely to lose again and, after winning, it is more likely to be victorious (Chase, 1974; Hsu et al., 2006; Franz et al., 2015). The build-up of a dominance hierarchy via the winner-loser effect is referred to as the “self-organization hypothesis” (Hogeweg and Hesper, 1983; Dugatkin, 1997; Hemelrijk, 2000). The model DomWorld comprises individuals that group, compete and experience the self-reinforcing effects of conflict outcomes. In it, males and females are identical in all respects except in two aspects of their fighting ability (reflecting prior attributes). Firstly, males start with a higher initial fighting power (dominance) than females, though fighting ability subsequently changes over time via the winner-loser effect. Secondly, the aggression of males is more intense, and thus has more impact, than that of females. For instance, being hit or trampled by a male involves more physical damage to the victim than by a female. The model shows that despite these favorable prior-attributes for males, the winner-loser effect may result in females becoming dominant over a few males in species where aggression is fierce. Intense or fierce aggression involves behavior, such as chasing, hitting and biting, as shown, for instance, by both sexes in rhesus monkeys, *Macaca mulatta* (Cheney and Seyfarth, 1990; Hemelrijk et al., 2008; Thierry et al., 2008). Female dominance over males happens in the model because the large impact of the winner-loser effect causes some males and females to lose much of their fighting ability and others to gain a lot. Thus males may drop in their fighting ability below certain females, without necessarily having had a direct conflict with these females. When aggression is mild, female dominance is less likely to emerge because the impact of winning and losing fights is minor and therefore the individuals neither rise, nor sink much in their fighting ability and thus, their rank. Therefore, when aggression is mild and females start with a lower fighting capacity than males, the females remain subordinate. Tonkean macaques (*M. tonkeana*) are an example of a primate species which exhibit mild aggression during conflicts (such as staring), and therefore the outcomes of their fights have only a small impact (Thierry et al., 2008).

For a species with high intensity of aggression, the model DomWorld has three predictions. First, the higher the proportion of males in the group, the more dominant females become over males. This happens only when the intensity of aggression of males is higher than that of females, as is usual in primates with male-biased sexual dimorphism (Albers and de Vries, 2001; Clutton-Brock and Huchard, 2013). Second, in groups with more males greater dominance of females over males through greater subordinance of males in the model is due to the greater relative frequency of male-male fights (which have high impact compared to fights with females). Third, in groups with a greater proportion of males, females are expected to win fights against males more often as proportion of their fights with all adults. Thus, the first prediction concerns a general pattern and the second and third one are associated processes. The relationship between the proportion of males in the group and female dominance over males has subsequently been tested and confirmed in a few groups of rhesus monkeys, and in a small dataset combining groups of several species of despotic macaques with intense aggression (Hemelrijk et al., 2008). Yet, when combining data from several species, the correlation is confounded by the effects of species-specific differences in sexual dimorphism. Thus the correlation is best studied among groups of a single species. Additionally, there is an indication for a similar process in humans. Here, “female influence” on a collective decision was taken as a proxy for female dominance and it was shown that this increases with proportion of men in the group (Stroebe et al., 2016).

These positive associations between proportion of males on the one hand, and on the other hand, female dominance, proportion of fights among males and proportion of victories of females over males, may well be a general phenomenon among groups within species, because the model DomWorld involves only four phenomena (processes and traits) and these are probably present in many mammals: First, the self-reinforcing effects of winning and losing fights (Hsu et al., 2006); second, strong intensity of aggression (with clear impact of an outcome of a fight on dominance); third, stronger intensity of aggression in males than females (note that most mammals have male-biased sexual dimorphism (Clutton-Brock, 2016) and therefore probably stronger aggression intensity in males); and fourth, a range of different sex ratios of groups (so that we can study a sufficiently large range of adult sex ratios).

To investigate the generality of these dynamics in dominance between the sexes, in the present study we investigate them in the vervet monkey (*Chlorocebus pygerythrus*). We chose vervet monkeys as this species shows fierce aggression (Cheney and Seyfarth, 1990) and some degree of female dominance over males (Struhsaker, 1967; Smuts, 1987; Hemelrijk et al., 2008; Young et al., 2017). Although the self-reinforcing effects of winning and losing fights have not yet been studied empirically in vervet monkeys, it is likely that they operate in this species, because the winner-loser effect has been shown in many taxa (Hsu et al., 2006) including primates, namely rhesus monkeys (*M. mulatta*; Mendoza and Barchas, 1983; Neumann et al., 2011; Snyder-Mackler et al., 2016), crested macaques (*Macaca nigra*), yellow baboons (*Papio cynocephalus*), anubis baboons (*Papio anubis*; Franz et al., 2015), and chimpanzees (*Pan troglodytes*; Newton-Fisher, 2017). Moreover, in vervet monkeys it is likely that males are more intense in their aggression than females because of their sexual dimorphism. Further, we have long-term data on conflicts collected from 2011 until and including 2017 in four groups of vervet monkeys living under natural conditions at the Inkawu Vervet Project (IVP), in Mawana Game Reserve, KwaZulu Natal, South Africa.

In line with the self-organization hypothesis, we predict that processes of self-organization in groups of vervet monkeys imply that a higher proportion of males in a group will result in an increase of (a) female dominance over males, (b) proportion of male-male fights, and (c) proportion of victories of females over males.

In case greater female dominance in groups with a higher proportion of males is found, we also examine two alternative hypotheses for the self-organization hypothesis namely whether this pattern may result from (1) higher frequencies of support received by females from either sex in fights against males, the social support hypothesis (Smuts, 1987) and from (2) lowered aggression of males to females as a kind of sexual exchange, when competing with more males (sometimes labeled the docile male hypothesis; Surbeck and Hohmann, 2013).

Besides, we test the relationship between female dominance over males and proportion of males in the group not only with data in Mawana but also with data from the literature on two other sites, Masai-Amboseli Game reserve, south-central Kenya, East Africa (Struhsaker, 1967) and the Samara Private Game Reserve, South Africa (32°22’S, 24°52’E; Young et al., 2017).

## Materials and Methods

### Species

In vervet monkeys, females are philopatric and males usually migrate at around 4–5 years of age. Female rank is influenced by kinship (the youngest daughter will usually attain the rank just below her mother), whereas an adult male’s rank depends on his own ability to win conflicts against other adult males (Cheney and Seyfarth, 1990) and may also be strengthened by social positive relations with females (as reflected in grooming and proximity; Young et al., 2017).

As to sexual dimorphism, body weight of males is on average 1.4 times that of females in the wild (males weigh on average 5.7 ± 0.07 kg and females 4.1 ± 0.05 kg; Turner et al., 2018), males are significantly more muscular than females and adult canine lengths of males is about 1.3 times that of females (Bolter and Zihlman, 2003).

### Data Collection in Mawana Game Reserve

Behavioral data were collected as part of the IVP in Mawana Game Reserve, KwaZulu Natal, South Africa between January 2011 and December 2017 in four neighboring groups of wild vervet monkeys, named Ankhase, Baie Dankie, Kubu, and Noha. The home ranges of all groups differ in their spread of vegetation and within each home range there are areas of cluttered vegetation, for instance close to the river, and areas of more spread out vegetation, for instance large areas of acacia.

Group size included typically about 30 individuals in total with on average 13.8 adults and ranged between 7 to 24 adults. We confined our analyses to adults; a female was considered to be adult after she had given birth, and a male after his first dispersal to another group.

The monkeys were habituated to human presence from 2010 onward. Data collection on a group started after human observers could approach each monkey within 10 meters. Data were collected during several days a week continuously throughout the year. Per group we collected data for the following hours and days, in group Ankhase 9763 h during 1553 days, in Baie Dankie 12,044 h during 1,707 days, in Noha 12,141 h in 1,729 days, and in group Kubu 5,367 h in 937 days. Observers moved throughout the group in order to collect scan and focal data on all group members and to reduce bias toward particular individuals.

Data on conflicts were recorded with *ad libitum* sampling while observers were collecting scan and focal sampling data, while habituating groups, or conducting field experiments. Conflicts were defined by the occurrence of one or more of the following elements in a social interaction: “hit,” “bite,” “grab,” “stare,” “attack,” “chase,” “displacement,” “steal food,” “hand on head,” and “aggressive call.” For each conflict, the following was recorded: the time of the event, the identity of the opponents, the winner and the identity of the group. To determine the dominance position of an individual in the group, we used only dyadic conflicts ignoring polyadic conflicts that involved more members. An individual was considered to have won a conflict, if its last behavior in the conflict was aggressive (as defined above) *and* the last behavior of its opponent was submissive (“avoid,” “jump aside,” “crawl,” “leave,” “retreat,” “flee,” and “scream”). If either opponent’s last behavior was ambiguous (e.g., “undetermined vocalization”), its directly preceding behavior was used instead to determine the outcome of the conflict. Still, the behavior of one opponent had to be clearly aggressive, *and* that of the other clearly submissive, for the conflict to be included. In the case of an ambiguous outcome, conflicts were discarded for determining dominance. The intensity of each conflict was recorded as either severe (hit, bite, chase, grab, or steal food) or mild (stare, displace, and aggressive call). In our analysis of the social support hypothesis, we defined support in a conflict, as the case in which a third individual joined in a fight between two others by attacking one of the opponents. All researchers were trained and tested to reliably identify all individual monkeys.

### Data and Analysis of Mawana Game Reserve

We put conflicts in matrices per group per year, with the identity of winners listed in rows and of losers listed in columns. We confined our analyses to dyadic interactions among adults and studied in total 159 adults during 37,083 observation hours. Data on adults were included only if they had been present in the group for at least half a year. Thus, in the case of females, because they are adult after giving birth, females were included only if they had given birth to their first offspring more than 6 months ago or longer and immigrant males were included after they had been in the group for 6 months or longer. Conflict matrices (referred to as group-year points) were used in our analysis only when (1) at least 50 conflicts were recorded in a given year, and (2) the conflicts had been collected throughout an entire year (excluding data collected during a shorter period). We chose a period of a full year rather than half a year, in order to reduce the effect of the short period of hierarchical instability that happened after the single migratory period that took place each year. In total we recorded 3123 conflicts over 16 group-year points collected for four groups during 7 years. On average individuals were recorded to participate in 28 conflicts per year (range: 7 to 153).

We used the total number of dyadic, agonistic interactions among all adults per group and year (excluding cases of support in conflicts) to determine the linear dominance hierarchy. We ranked individual adults in a group according to their fraction of winning fights of all fights with each partner averaged over all interaction partners with whom they had been in conflict (discarding group members with whom they had no interactions), the so-called average dominance index, ADI (Hemelrijk et al., 2005). Thus, this index controls for some dyads having more interactions than others. A higher value implies greater dominance of an individual. We choose this method because of its robustness, as compared to other measures such as IS&I and Netto (Hemelrijk et al., 2005). Its outcome and robustness are the same as that of David Score, provided that missing values are taken care of properly when calculating the David Score.

We quantified relative female dominance in a group (the female dominance index, FDI) as the proportion of males over which the females were dominant on average (Hemelrijk et al., 2008). Using the dominance hierarchy of both sexes based on the average dominance index, ADI we summed over all females the number of males that were ranking below each female (and in the case of a tie, males were counted as half) and divided this by the maximum number of males that could have ranked below all females (which equals the number of females multiplied by the number of males; Hemelrijk et al., 2003). This FDI over males ranges from 0 (all females are subordinate to all males) to 1 (all females are dominant over all males; Hemelrijk, 1999; Hemelrijk et al., 2008). We investigated whether the proportion of adult males of the total number of adults in the group was related to (a) the female dominance index, FDI, (b) the proportion of fights of males with other males of all their fights with adults, and (c) proportion of fights won by females of all their fights with adults (*n* = 16 group-year points, Table 1).

**TABLE 1:** Information on the reserve, the group-name, number of adults of each sex, male proportion, female dominance, and individual rankings of both sexes per group per year. * means the adjacent individuals have the same average dominance index, ADI.

**Reserve**	**Group**	**Year/ Period**	**Male#**	**Female #**	**Male Pro-portion**	**Female dominance**	Ranking from high to low (*means the adjacent individuals have the same ADI)
Mawana	Ankhase	2011	2	5	0.29	0	M, M, F, F, F, F, F
Mawana	Ankhase	2012	6	6	0.5	0.58	M, F, F, M, F, F, M, M, F, F*, M*, M*
Mawana	Ankhase	2013	4	9	0.31	0.51	F, M, F, F, M*, F*, F, F, M, F, F, M, F
Mawana	Baie Dankie	2011	4	8	0.33	0.25	M, M, F, F, F, M, F, F, M, F*, F*, F*
Mawana	Baie Dankie	2012	4	12	0.25	0.46	F, M, F, M, F, F, F, F, F*, F*, F, M, F, M, F, F,
Mawana	Baie Dankie	2013	4	11	0.27	0.43	M, F, F, F, F*, M*, F*, F, F, M*, F*, M*, F, F*, F*
Mawana	Baie Dankie	2014	8	7	0.53	0.42	F, M, M, M*, M*, F, F, M*, F*, M, F, M*, F*, F*, M*
Mawana	Baie Dankie	2015	6	11	0.35	0.38	F, M, F, M*, M*, M, F, F, F, F, F*, F*, M, F, F*, F*, M*
Mawana	Baie Dankie	2016	6	11	0.35	0.27	M, M, M, F, F, F, M* F*, F*, F, F, M, M, F, F, F*, F*
Mawana	Baie Dankie	2017	12	12	0.5	0.40	F, F, M, M, M, M, F, M, M*, F*, M, M, F, F, M, F, F, M, F, M, F, F, F, M
Mawana	Kubu	2017	1	5	0.17	0	M, F, F, F, F, F
Mawana	Noha	2011	1	9	0.1	0	M,F, F, F, F, F, F, F, F, F
Mawana	Noha	2012	5	10	0.33	0.52	F, F, M, F, F, M, M, F, F, M, F, F, F, F, M
Mawana	Noha	2013	5	11	0.31	0.45	F, F, M, F, M, M, F*, F*, F, F, M, F, F, F, M, F
Mawana	Noha	2014	7	11	0.39	0.27	F*, M*, F, M, M, F*, M*, M*, F, F*, F*, M*, M+, F+, F, F*, F*, F*
Mawana	Noha	2016	2	6	0.25	0.08	M, F, M, F, F, F, F, F
Samara	PT	1	10	9	0.53	0.28	M, M, M, F, M, M, F, M, M, F, F, F, M, F, F, M, M, F, F
Samara	PT	2	10	9	0.53	0.32	M, M, F, M, M, F, F, M, M, M, F, M, F, F, F, M, M, F, F
Samara	PT	3	7	12	0.37	0.44	F, M, F, F, M, M, F, F, F, M, F, M, M, F, F, F, M, F, F
Samara	PT	4	6	11	0.35	0.52	F, F, F, F, M, F, M, F, M, M, M, F, M, F, F, F, F
Samara	PT	5	6	10	0.38	0.47	F, F, M, F, M, M, F, M, F, F, M, F, F, F, F, M
Samara	PT	6	4	10	0.29	0.18	M, F, F, M, M, F, M, F, F, F, F, F, F, F
Samara	RBM	1	13	12	0.52	0.26	F, F, M, F, M, M, M, M, M, M, M, M, M, M, M, F, F, M, F, F, F, F, F, F, F
Samara	RBM	2	19	13	0.59	0.26	M, M, M, F, F, M, F, M, M, M, M, M, M, M, M, M, F, M, M, M, F, F, M, F, F, M, F, M, F, F, F, F
Samara	RBM	3	15	13	0.54	0.53	F, F, F, M, F, F, F, F, M, M, M, M, M, M, M, M, M, M, M, M, F, M, M, F, F, F, F, F
Samara	RBM	4	19	13	0.59	0.68	M, M, F, F, F, F, M, M, M, F, F, M, F, M, F, F, F, M, F, M, F, M, M, M, M, M, M, M, M, F, M, M
Samara	RBM	5	16	13	0.55	0.51	F, F, M, F, F, M, M, F, M, F, M, M, M, M, M, F, M, M, F, F, M, M, M, M, F, F, M, F, F
Samara	RBM	6	13	13	0.5	0.5	F, F, M, M, M, M, F, M, M, F, M, F, F, M, F, F, F, M, F, F, F, M, F, M, M, M
Samara	RST	1	15	21	0.42	0.41	F, F, M, M, F, F, M, F, F, M, M, F, M, M, M, M, M, M, F, F, F, F, M, F, F, F, F, F, M, F, F, F, M, F, M, F
Samara	RST	2	12	15	0.44	0.47	F, F, M, M, M, F, F, F, M, F, F, M, M, F, M, M, F, F, M, M, M, F, F, F, M, F, F
Samara	RST	3	10	15	0.4	0.23	F, M, M, F, M, M, F, M, F, M, M, F, M, F, M, F, M, F, F, F, F, F, F, F, F
Samara	RST	4	13	17	0.43	0.53	M, M, F, F, M, F, F, F, F, F, F, F, M, F, M, M, M, F, M, M, F, M, F, F, M, M, F, M, F, F
Samara	RST	5	13	16	0.45	0.45	F, F, F, M, F, F, M, M, M, M, M, F, M, F, F, F, M, M, M, F, M, F, M, F, M, F, F, F, F
Samara	RST	6	14	16	0.47	0.43	F, M, M, F, F, F, M, F, M, F, M, M, F, M, M, M, F, F, M, M, F, F, M, M, F, M, F, F, F, F
Amboseli	1530		2	3	0.4	0.17	M, F, M, F, F
Amboseli	P		3	4	0.43	0.38	M, F*, F*, M*, F, F*, M*

*^∧^Although there were six females resident in Kubu in 2017, one did not participate in any conflict was therefore excluded from further analysis. ^∗^These individuals had tied values for their dominance index (ADI) with one or more of the adjacent individuals also marked with a ^∗^.*

Further, to investigate the social support hypothesis, by testing for a relationship between proportion of males in a group and how often females received support from males in dyadic conflicts against other males, we calculated for each group-year, the average proportion of male-female dyadic conflicts in which females were supported by another male. We studied the relation between these averages and the proportion of males in the group. We removed two groups with only one male (Noha in 2011 and Kubu in 2017, Table 1) as male support against males was here impossible) leaving 14 group-year points. We similarly investigated support received from females by females in their fights against males.

To test the docile male hypothesis, we examined whether the intensity and frequency of aggression from males toward females was reduced in periods of stronger competition for access to females. We compared intensity and frequency of aggression of males to females during the mating season (from April until and including July, 4 months) to the rest of the year (8 months).

### Data and Analysis From Studies in Amboseli and Samara Private Game Reserve

**FIGURE 1:**
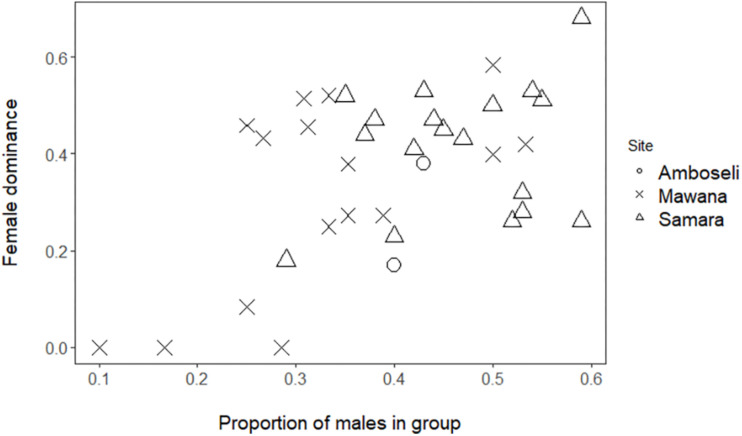
Female dominance, FDI, versus proportion of males (only adults are concerned) in Mawana game reserve (symbol: cross), Amboseli (symbol: circle; Struhsaker, 1967), and Samara Private game reserve (symbol: triangle; Young et al., 2017).

We determined the dominance hierarchy and female dominance index, FDI in two groups in Amboseli using the same analyses as in Mawana. Data in Amboseli were collected in a study of one year shown in Tables 6 and 7 in Struhsaker (1967). We also determined the FDI in Samara private game reserve using data of three groups collected during a study of 3.5 years on three groups and shown in Figure 1 of the article by Young and colleagues (Young et al., 2017; Table 1).

### Statistics

#### Self-Organisation Hypothesis

To test whether he female dominance index, FDI could be predicted by proportion of males in Mawana, we used a Generalized Linear Mixed Model (GLMM), assuming a beta-binomial distribution for the total number of cases that individual males were subordinate to each of the females, summing them over all females of a group (using *N* = 16 group-year combinations). The choice for this model is motivated by the fact that the female dominance index in a group (FDI) is the sum of the total number of males dominated by each of the females (thus the same male may be counted several times if it is dominated by several females) divided by the total number of males that could have been dominated by each female, summed over all females. Note that this equals the average of the fraction of males subordinate to each female. The beta-binomial distribution (instead of the ordinary binomial distribution) is used to handle possible overdispersion, as there is no reason to believe that the variation of the fractions will be of binomial origin only. In the GLMM we related the female dominance index, FDI in a group to the proportion males, using a logit link function, as is common for binary data. We introduced crossed random group effects for groups and years into the model to handle repeated observations of the same group over different years and to account for differences between years (possibly reflecting climatological effects).

Using the extended dataset of three sites (Mawana, Samara, and Amboseli), we analyzed the relationship between the female dominance index as the response variable (FDI) and explanatory variables proportion of males in the group and sites (together forming the fixed part of the model) and crossed random effects of groups and years, using a betabinomial GLMM (Table 1, *N* = 36 group-year combinations).

We tested the associated processes related to the proportion of fights among males and the proportion of victories of females over males only in Mawana: The response variables, proportion of fights among males of all of fights by males and the proportion of fights won by females over males, were related to the proportion of males in a group again using betabinomial GLMMs (*N* = 16 group-year points). The random part of the GLMMs consisted of crossed random effects of years and groups.

### Alternative Hypotheses

We tested the following alternative hypotheses only in Mawana.

In case of the social support hypothesis, the response variables proportion of support of fights with males received from males by females (*N* = 16 group-year points) and the support proportion received from females by females (*N* = 16 group-year points) were analyzed with GLMMs, as described for female dominance.

To test the docile male hypothesis, we analyzed the number of conflicts of males with females per male aggressor per month in three ways, (1) in total (*N* = 221 male-month combinations, 39 males), (2) the mild conflicts (*N* = 194 male-month combinations, 38 males), and (3) severe conflicts (*N* = 80 male-month combination, 28 males) separately. We used GLMMs with a truncated negative binomial distribution, fixed effect for mating season (Y/N) and crossed random effects for group and year and individual aggressor nested within group and year. Because only males with at least one fight were considered, the truncated negative binomial distribution, which assumes that counts ≥1, was used as probability distribution for the numbers of conflicts.

### General Information on Statistics

All GLMM models were fitted using the glmmTMB package (Brooks et al., 2017) of *R* (version3.6.1, R Core Team, 2019). In the Supplementary Material we give statistics on model diagnostics [goodness of fit statistics based on simulated residuals as described in the R-package DHARMa (Hartig, 2019)] and model performance [omnibus likelihood ratio tests comparing the fitted model with the null model, and pseudo *R*^2^ based on likelihoods using R-package MuMIn (Barton, 2019)].

Since the conceptual details of GLMMs are not as clear as those of correlations and to indicate the robustness of our results, we mention that we also have tested these patterns with the more old-fashioned methods of correlations (Pearson and Kendall, where suited) and Bonferroni Holm methods, ignoring the repeated observations on some individuals that returned in different group-year points. This has led qualitatively to the same results, see Supplementary Material.

## Results

### The Self-Organisation Hypothesis

Female dominance index over males, FDI, in wild vervet monkeys in Mawana Game reserve had an average value of 0.31 (standard deviation = 0.20, min = 0, and max = 0.58) which resembles the values for the Samara private game reserve with an average value of 0.42 (standard deviation = 0.13, min = 0.18, and max = 0.68). Female dominance, FDI, in both reserves is higher than the average value of 0.27 found for the two groups in Amboseli (groups 1530 and Struhsaker, 1967; Hemelrijk et al., 2008; for group size, composition, and ranks, see Table 1).

We showed that female dominance over males, FDI, is significantly positively associated with the proportion of males in the group in Mawana (GLMM, 4 groups, 16 group-year-points, regression coefficient β = 3.6, SE 1.2, *z*-value 3.0, and *P* = 0.002; Figure 1). Although in the data of private game reserve Samara separately, the same association was positive, but non-significant (GLMM, 3 groups, *n* = 18 group by half year records, β = 1.23, SE = 1.49, *z*-value = 0.83, and *P* = 0.41), when we combined the data of the three sites, Mawana, Amboseli and Samara, the FDI and proportion of males were significantly associated [GLMM, 9 groups (4 in Mawana, 3 in Samara, 2 in Amboseli), 36 group-year points, β = 2.6, SE = 0.95, *z*-value = 2.21, and *P* = 0.0064, Figure 1]. No significant differences in female dominance, FDI, corrected for proportion of males were found between sites (same data set, *N* = 36, likelihood ratio test *X*^2^ = 0.14, and *P* = 0.93).

**FIGURE 2:**
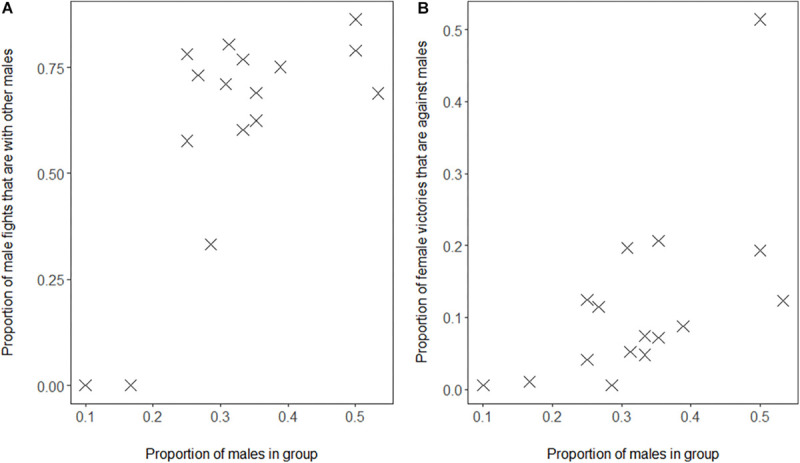
Proportion of males in Mawana versus **(A)** proportion of fights of males with other males out of all fights of males with adults, **(B)** proportion of fights won by females against males out of all fights of females with adults. Note that also after removing the outlier in **B**, our result is significant (slope = 5.1, *z*-value = 3.49, and *P* = 0.0005).

We confirmed the associated processes based on the self-organization hypothesis, namely that in groups with a higher proportion of males, (a) males fight relatively more with other males as proportion of their interaction with both sexes (Figure 2A, GLMM, 4 groups, 16 group-year points, regression coefficient β = 9.27, SE 2.27, *z*-value 4.08, and *P* = 0.00005) and (b) females win conflicts with males more often as a proportion of their winning conflicts with either sex (Figure 2B, GLMM, 4 groups, 16 group-year points, 126 females, regression coefficient β = 6.96, SE 1.66, *z*-value 4.20, and *P* = 0.00003).

### Alternative Hypotheses

#### The Social Support Hypothesis

**FIGURE 3:**
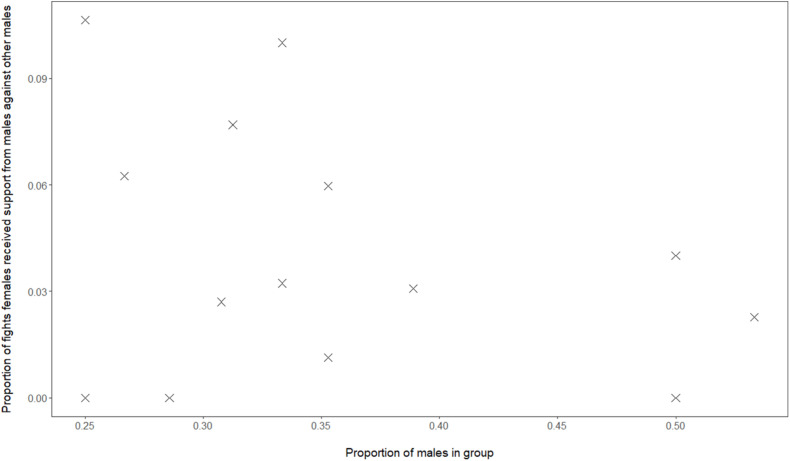
Data from vervets in Mawana regarding the proportion of males versus the proportion of fights of females against males in which females receive support from males.

Although, in our data of Mawana, we have calculated the dominance indices using only dyadic interactions, the social support hypothesis cannot be excluded, namely that in groups with a greater proportion of males, female dominance over males, FDI, may be higher due to females receiving more support from either sex, when females are in conflict with a male. When studying support in fights received by females, we do not find a correlation between proportion of males in the group and proportion of female-male conflicts in which the female was supported by another male (GLMM, 4 groups, 16 group-year points, regression coefficient β = 1.86, SE 2.30, *z*-value 0.81, and *P* = 0.42, see Figure 3). However, when the proportion males in the group was higher this was positively associated with a greater proportion of female-male conflicts in which the female was being supported by another female (GLMM, 4 groups, 16 group-year points, regression coefficient β = 56, SE 1.61, *z*-value 2.83, and *P* = 0.005).

### Docile Male Hypothesis

**FIGURE 4:**
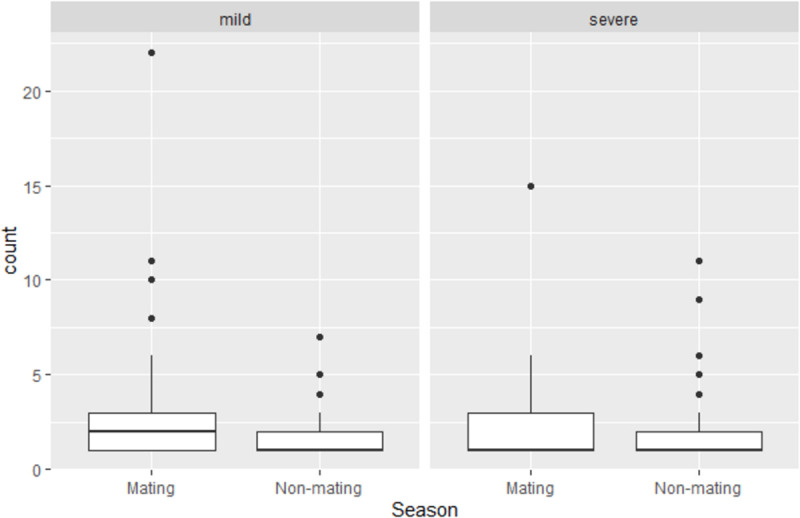
Boxplots of number of severe and mild conflicts initiated from males to females per month per male in the mating season (from April till and including July) and in the rest of the year.

The docile male hypothesis was not supported by our data, neither when analyzing data on all conflicts, nor when analyzing mild and severe conflicts, separately. Note that in our 16 group-year sample, 3675 conflicts recorded were coded as mild, and 726 as severe. Based on the GLMMs, the total aggression of males to females per month was even lower (though not-significantly so) during the non-mating season when considering all conflicts (regression coefficient for non-mating season β = −0.18, SE = 0.23, *z*-value = −0.81, and *P* = 0.42), the number of mild conflicts (β = −0.47, SE = 0.29, *z*-value = −1.62, and *P* = 0.10) and the number of severe conflicts (β = −0. 35, SE = 0.36, *z*-value = −0.97, and *P* = 0.33, Figure 4).

## Discussion

We confirmed in wild vervet monkeys the theoretical prediction (Hemelrijk et al., 2008), that higher female dominance over males is associated with a higher proportion of males in the group (in Mawana, and in the combination of three sites in Africa, namely Mawana, Samara, and Amboseli). We additionally confirmed that the higher the proportion of males in the group, the more often males interacted agonistically with other males relative to interacting with all adults and the more often females won fights against males versus against all adults. This confirms the three predictions of the model DomWorld and indicates that self-reinforcing effects of winning and losing fights may underlie dynamics of dominance between the sexes in vervet monkeys.

Further, we found neither an indication that, in groups with a higher proportion of males, females become higher in rank by receiving more support from males in fights with other males, the social support hypothesis (Smuts, 1987; Smuts and Smuts, 1993; Parish, 1994; Setchell et al., 2006; White and Wood, 2007), nor that females increase rank, because males are reducing aggression to females for getting access to them, as suggested in the docile male hypothesis (Surbeck and Hohmann, 2013). Whereas males experience the strongest competition for access to females in the mating season, in this season they do not reduce aggression toward females compared to outside this season. We find, however, that in groups with a higher proportion of males, females are receiving more support from females. This does not necessarily mean that females become higher in rank due to the support they received from other females. Instead, they may already be higher in rank than males they support against. Their high rank relative to males may have arisen by the self-reinforcing effects of winning and losing fights. In such a case, female support itself may be a side effect of females being already higher in rank relative to males and thus, experiencing less risk in joining other females against males in groups with more males. We gave a similar argument for the higher frequency of support among females in bonobos versus chimpanzees (Hemelrijk, 2002); since, compared to female chimpanzees, female bonobos are already higher in rank than their male group members, they experience less risk to join in fights of other females against males.

Two further alternative explanations for finding the positive association between female dominance over males and proportion of males in the group are:

First, rather than being a consequence of group composition, female dominance over males causes the composition of the group, meaning that in some groups, females of high dominance permit more males to enter the group, because males are not aggressive toward anyone. However, this can be excluded in Mawana, because males are on average more aggressive than females in 15 out of our 16 group-year points (Wilcoxon signed-rank test, *N* = 16, *V* = 4, and *p*-value = 0.0002).

Second, in groups with more males, males may compete more for sexual access to females. As females may here be a limiting resource, this may increase the female’s value and thus, dominance relative to males (Goodall, 1986). In line with our self-organization hypothesis, this would imply that the total frequency of male-male aggression is higher in groups with more males, which we confirm in Mawana and so we are unable to exclude this hypothesis.

Although female primates have usually been considered to rank below males because of the smaller size of their body and canines, some dominance by females over males has already been found in vervet monkeys in Amboseli and in Samara (Struhsaker, 1967; Hemelrijk et al., 2008; Young et al., 2017). We have confirmed this in a new site, Mawana. We show that, combining data of the three sites, the degree of female dominance over males, FDI, in wild vervet monkeys is on average 0.36 (SE = 0.03), thus, below co-dominance of 0.5. In all three sites combined, the relation between proportion of males and the index of female dominance over males, FDI, was positive and significant. The non-significant but positive trend in the data of private game reserve Samara separately may be related to the smaller range of sex ratios over which this correlation was studied in Samara (0.1 to 0.5 in Mawana and 0.3 to 0.6 in Samara).

In future work it would be interesting to quantitatively study the degree of female dominance over males in many species of mammals with male-biased sexual dimorphism (including and beyond primates) living in multi-male groups with different proportions of males and study how these species differ in the relation between proportion of males and degrees of female dominance over some males. We particularly expect the association to be found for species for which some female dominance over males has been reported despite male-biased sexual dimorphism. In primates, for instance, these are bonobos (Vervaecke et al., 2000), capuchin monkeys (Izawa, 1980), several species of macaques (Rhine et al., 1989; Hemelrijk et al., 2008), common chimpanzee (Hemelrijk and Ek, 1991), common squirrel monkey (Masataka and Biben, 1987), and the gray langur (Sommer et al., 2002). It would also be interesting to see whether the adult sex ratio depends on certain environmental conditions. As to the winner-loser effect, it should be specifically tested in vervet monkeys, like it has been in baboons (Franz et al., 2015) and other species.

We explicitly note that, our theory based on DomWorld, was not developed for species (almost) lacking sexual dimorphism in body size and aggression intensity, such as hyenas and lemurs, and having special adaptations related to female dominance such as masculinized genitals or high levels of testosterone (von Engelhard et al., 2002; Wagner et al., 2007).

According to the theoretical study, DomWorld, the positive relation between proportion of males and female dominance over males should be absent (or weaker) in species with aggression that is mild, for example, in tonkean macaques and in crested macaques (Hemelrijk et al., 2008). What precise phases of different degrees of female and male dominance pass through, when sex ratio changes in groups, should be studied experimentally in detail similarly to the transitivity analyses of Lindquist and Chase (2009) and be related to the winner loser effect and to spatial structure (Hemelrijk et al., 2017).

Note that our explanation for different degrees of female dominance over males is integrative in the sense of considering a combination of traits (the winner-loser effect, species-specific intensity of aggression, higher intensity of aggression by males than females and a range of sex ratios of a group), and their consequences. This integrative aspect is typical for explanations based on self-organization.

Along the lines of studies testing the effects of self-organization in complex systems, we conclude that inter-sexual dominance in vervet monkeys probably depends on the winner-loser effect, because it depends on the adult sex ratio of a group. In order to establish the winner-loser effect convincingly, however, is beyond the scope of this paper. For this further studies are needed examining time-series in aggressive interactions in empirical data (as done, for instance, by Franz et al., 2015). In general, based on our results in vervet monkeys, we urge future empirical studies of intersexual dominance to also take sex-ratio and fierceness of aggression into account.

## Data Availability Statement

The datasets generated for this study are available on request to the corresponding author.

## Ethics Statement

The study was approved by the relevant authority (Ezemvelo KZN Wildlife, SouthAfrica) and by the funders. The study conforms with the ASAB/ABSguidelines for the care and use of animals. We used non-invasive observational methods of data collection on animals in their natural habitats, and all individuals were habituated to human observers.

## Author Contributions

CH conceived the idea of this manuscript and wrote the main part of the manuscript, and contributed to the statistical analysis, figures and tables. EW designed the data collection and trained the team to it; coordinated the field site; funded most of the data collection; contributed to the data analyses; and wrote part of the manuscript and commented on it. MW extracted the data, conducted the statistical analyses, contributed to the writing of parts of the materials and methods and results. JB collected some of the data, contributed to the data analyses, and contributed to the writing of part of the materials and methods and results. GG contributed to the data analyses and their description in the materials and methods and results.

## Conflict of Interest

The authors declare that the research was conducted in the absence of any commercial or financial relationships that could be construed as a potential conflict of interest.
