# Corrigendum: Dynamics of Intersexual Dominance and Adult Sex- Ratio in Wild Vervet Monkeys

**DOI:** 10.3389/fpsyg.2020.598699

**Published:** 2020-10-28

**Authors:** Charlotte Korinna Hemelrijk, Matthias Wubs, Gerrit Gort, Jennifer Botting, Erica van de Waal

**Affiliations:** ^1^Groningen Institute for Evolutionary Life Sciences, University of Groningen, Groningen, Netherlands; ^2^Department of Ecology and Evolution, University of Lausanne, Lausanne, Switzerland; ^3^Inkawu Vervet Project, Mawana Game Reserve, Kwazulu Natal, South Africa; ^4^Biometris, Wageningen University & Research, Wageningen, Netherlands

**Keywords:** the winner-loser effect, dominance hierarchy, fierceness of aggression, female dominance over males, adult sex-ratio, vervet monkeys

In the original article, there was a mistake in Table 1 as published. The values for the female dominance (index) and dominance ranking from high to low of adults for all group-year points in Mawana were incorrect, apart from in Baie Dankie 2013 where only the ranking of adults was incorrect and in Noha 2011 where all information was correct. The number of females was incorrect in Kubu 2017 and so was the proportion of males; in Noha 2012 the number of males was incorrect and therefore the proportion of males was incorrect. Data on Baie Dankie 2014 were added. Data on Noha 2015 are omitted. The corrected Table 1 appears below.

**Table 1:** Information on the reserve, the group-name, number of adults of each sex, male proportion, female dominance, and individual rankings of both sexes per group per year. ^*^ means the adjacent individuals have the same average dominance index, ADI.

**Reserve**	**Group**	**Year/ Period**	**Male#**	**Female #**	**Male Pro-portion**	**Female dominance**	Ranking from high to low (^*^means the adjacent individuals have the same ADI)
Mawana	Ankhase	2011	2	5	0.29	0	M, M, F, F, F, F, F
Mawana	Ankhase	2012	6	6	0.5	0.58	M, F, F, M, F, F, M, M, F, F^*^, M^*^, M^*^
Mawana	Ankhase	2013	4	9	0.31	0.51	F, M, F, F, M^*^, F^*^, F, F, M, F, F, M, F
Mawana	Baie Dankie	2011	4	8	0.33	0.25	M, M, F, F, F, M, F, F, M, F^*^, F^*^, F^*^
Mawana	Baie Dankie	2012	4	12	0.25	0.46	F, M, F, M, F, F, F, F, F^*^, F^*^, F, M, F, M, F, F,
Mawana	Baie Dankie	2013	4	11	0.27	0.43	M, F, F, F, F^*^, M^*^, F^*^, F, F, M^*^, F^*^, M^*^, F, F^*^, F^*^
Mawana	Baie Dankie	2014	8	7	0.53	0.42	F, M, M, M^*^, M^*^, F, F, M^*^, F^*^, M, F, M^*^, F^*^, F^*^, M^*^
Mawana	Baie Dankie	2015	6	11	0.35	0.38	F, M, F, M^*^, M^*^, M, F, F, F, F, F^*^, F^*^, M, F, F^*^, F^*^, M^*^
Mawana	Baie Dankie	2016	6	11	0.35	0.27	M, M, M, F, F, F, M^*^ F^*^, F^*^, F, F, M, M, F, F, F^*^, F^*^
Mawana	Baie Dankie	2017	12	12	0.5	0.40	F, F, M, M, M, M, F, M, M^*^, F^*^, M, M, F, F, M, F, F, M, F, M, F, F, F, M
Mawana	Kubu	2017	1	5	0.17	0	M, F, F, F, F, F
Mawana	Noha	2011	1	9	0.1	0	M,F, F, F, F, F, F, F, F, F
Mawana	Noha	2012	5	10	0.33	0.52	F, F, M, F, F, M, M, F, F, M, F, F, F, F, M
Mawana	Noha	2013	5	11	0.31	0.45	F, F, M, F, M, M, F^*^, F^*^, F, F, M, F, F, F, M, F
Mawana	Noha	2014	7	11	0.39	0.27	F^*^, M^*^, F, M, M, F^*^, M^*^, M^*^, F, F^*^, F^*^, M^*^, M+, F+, F, F^*^, F^*^, F^*^
Mawana	Noha	2016	2	6	0.25	0.08	M, F, M, F, F, F, F, F
Samara	PT	1	10	9	0.53	0.28	M, M, M, F, M, M, F, M, M, F, F, F, M, F, F, M, M, F, F
Samara	PT	2	10	9	0.53	0.32	M, M, F, M, M, F, F, M, M, M, F, M, F, F, F, M, M, F, F
Samara	PT	3	7	12	0.37	0.44	F, M, F, F, M, M, F, F, F, M, F, M, M, F, F, F, M, F, F
Samara	PT	4	6	11	0.35	0.52	F, F, F, F, M, F, M, F, M, M, M, F, M, F, F, F, F
Samara	PT	5	6	10	0.38	0.47	F, F, M, F, M, M, F, M, F, F, M, F, F, F, F, M
Samara	PT	6	4	10	0.29	0.18	M, F, F, M, M, F, M, F, F, F, F, F, F, F
Samara	RBM	1	13	12	0.52	0.26	F, F, M, F, M, M, M, M, M, M, M, M, M, M, M, F, F, M, F, F, F, F, F, F, F
Samara	RBM	2	19	13	0.59	0.26	M, M, M, F, F, M, F, M, M, M, M, M, M, M, M, M, F, M, M, M, F, F, M, F, F, M, F, M, F, F, F, F
Samara	RBM	3	15	13	0.54	0.53	F, F, F, M, F, F, F, F, M, M, M, M, M, M, M, M, M, M, M, M, F, M, M, F, F, F, F, F
Samara	RBM	4	19	13	0.59	0.68	M, M, F, F, F, F, M, M, M, F, F, M, F, M, F, F, F, M, F, M, F, M, M, M, M, M, M, M, M, F, M, M
Samara	RBM	5	16	13	0.55	0.51	F, F, M, F, F, M, M, F, M, F, M, M, M, M, M, F, M, M, F, F, M, M, M, M, F, F, M, F, F
Samara	RBM	6	13	13	0.5	0.5	F, F, M, M, M, M, F, M, M, F, M, F, F, M, F, F, F, M, F, F, F, M, F, M, M, M
Samara	RST	1	15	21	0.42	0.41	F, F, M, M, F, F, M, F, F, M, M, F, M, M, M, M, M, M, F, F, F, F, M, F, F, F, F, F, M, F, F, F, M, F, M, F
Samara	RST	2	12	15	0.44	0.47	F, F, M, M, M, F, F, F, M, F, F, M, M, F, M, M, F, F, M, M, M, F, F, F, M, F, F
Samara	RST	3	10	15	0.4	0.23	F, M, M, F, M, M, F, M, F, M, M, F, M, F, M, F, M, F, F, F, F, F, F, F, F
Samara	RST	4	13	17	0.43	0.53	M, M, F, F, M, F, F, F, F, F, F, F, M, F, M, M, M, F, M, M, F, M, F, F, M, M, F, M, F, F
Samara	RST	5	13	16	0.45	0.45	F, F, F, M, F, F, M, M, M, M, M, F, M, F, F, F, M, M, M, F, M, F, M, F, M, F, F, F, F
Samara	RST	6	14	16	0.47	0.43	F, M, M, F, F, F, M, F, M, F, M, M, F, M, M, M, F, F, M, M, F, F, M, M, F, M, F, F, F, F
Amboseli	1530		2	3	0.4	0.17	M, F, M, F, F
Amboseli	P		3	4	0.43	0.38	M, F^*^, F^*^, M^*^, F, F^*^, M^*^

∧ *Although there were six females resident in Kubu in 2017, one did not participate in any conflict was therefore excluded from further analysis*.

* These individuals had tied values for their dominance index (ADI) with one or more of the adjacent individuals also marked with a *.

The authors apologize for this error and state that this does not change the scientific conclusions of the article in any way. The original article has been updated.

