# Supplementary material for: Dynamics of Intersexual Dominance and Adult Sex- Ratio in Wild Vervet Monkeys
Source: Front Psychol. 2020 May 14;11:839. doi: 10.3389/fpsyg.2020.00839 (PMC7240123; doi:10.3389/fpsyg.2020.00839)
Supplement: Supplementary file 1 [file Data_Sheet_1.docx]

**Supplementary Information**

Dynamics of intersexual dominance and adult sex- ratio in wild vervet monkeys

Hemelrijk C.K.^1, *^, Wubs M.^2 ,3^ Gort^4^ G., Botting J.^,3,5^,& van de Waal E.^2, 3,5^

^1^ Groningen Institute for Evolutionary Life Sciences (GELIFES), University of Groningen, Groningen, The Netherlands,

^2^ Department of Ecology and Evolution, University of Lausanne, 1015 Lausanne, Switzerland

^3^ Inkawu Vervet Project, Mawana Game Reserve, Kwazulu Natal, South Africa

^4^ Biometris, Wageningen University, Wageningen, The Netherlands

^5^ joint last authors

* Correspondence**:**Corresponding Author
c.k.hemelrijk@rug.nl

We here give

1. Extra information on GLMMs and
2. analyses based on correlations and combination methods.

I) Extra information on GLMMs:

Here we give extra details for GLMM’s for the nine relationships we tested in the main paper. We report some of the details of the GLMM’s, their performance statistics and their diagnostics. All GLMM’s were fitted using the glmmTMB package (Brooks et al, 2017) of R (version3.6.1, R Core Team 2019). Performance statistics are the omnibus likelihood ratio tests comparing the fitted model with the null model (containing only an intercept and no random effects), and pseudo R^2^ based on likelihoods using function r.squaredLR from the R-package MuMIn (Kamil Barton 2019). Diagnostics concern plots and goodness of fit statistics based on simulated residuals as described in the R-package DHARMa (Hartig 2019). We do not check separately for overdispersion, because, using the betabinomial and negative binomial distributions, as GLMM’s allow for binomial or Poisson overdispersion.

1) **Female dominance over versus proportion males in the group (Mawana)**

Using N=16 group-year combinations, a beta-binomial model was fitted to the response variable of female dominance. The fixed part of the model consisted of the proportion of males, and the random part consisted of crossed random effects of year and group.

The likelihood ratio test (LRT) of the full model versus the null model was highly significant (X^2^=16.1 on 2 d.f., P=0.0003; in this case we used the model without random group effect as the full model, because its variance component was estimated as zero, leading to an undefined likelihood).

The pseudo R^2^ was 0.64 for the fixed and the random part together. For the fixed part alone it was 0.46.

*
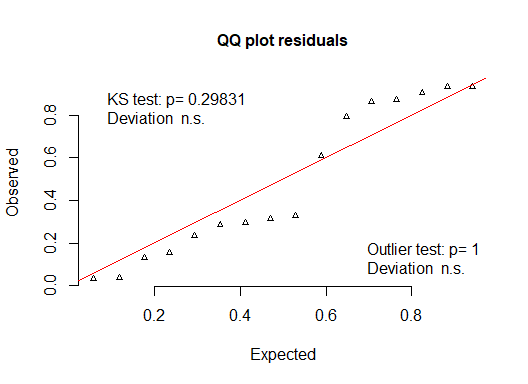
*Below we show the uniform QQ-plot based on 10.000 resamples from the DHARMa package. For a good fitting model the residuals should behave like a random sample from a uniform distribution. We confirm this with P=0.30 in the Kolmogorov-Smirnov (KS) test. The “Outlier test” checks in a different way whether residual patterns deviate from the uniform distribution and found no evidence either (KS test P=0.30; outlier test P=1, see plot below).

2) **Female dominance over males versus sites (Mawana, Samara, Amboseli) and proportion of males in the group**

Using N=36 group-year combinations a beta-binomial model was fitted to the response variable of female dominance. The fixed part of the model consisted of crossed effects of site (Mawana, Samara, Amboseli) and proportion of males in the group, and the random part consisted of crossed random effects of year and group.

The likelihood ratio test (LRT) of the full model versus the null model was significant (X^2^=13.3 on 5 d.f., P=0.02).

The pseudo R^2^ was 0.31 for the fixed and random part together, which is lower than for Mawana alone. For the fixed part alone it was 0.22.

The residual-based QQ-plot showed no evidence against the uniform distribution (KS test P=0.63; Outlier test P=1).


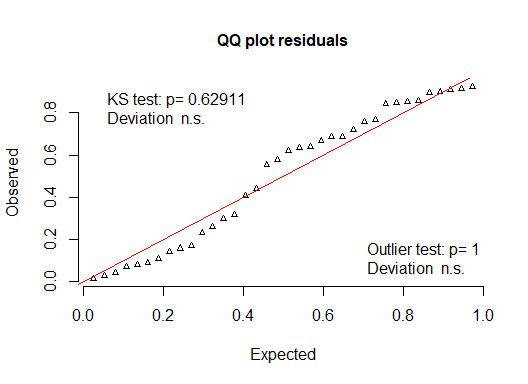


3**) Proportion of fights by males with males of fights with all adults versus proportion of males in the group**

Using N= 16 group-year combinations, we fitted a beta-binomial model to the proportion of male-male fights of all fights by males with adults. The fixed part of the model consisted of proportion males, whereas the random part consisted of crossed random effects of year and group.

The likelihood ratio test (LRT) of the full model versus the null model showed a significant result (X^2^=14.9 on 3 d.f., P=0.002).

The pseudo R^2^ was 0.61 for fixed and random part together. For the fixed part alone the pseudo R^2^ was 0.57.

The residual based QQ-plot shows no evidence against the uniform distribution (KS test P=0.91; Outlier test P=1).

**
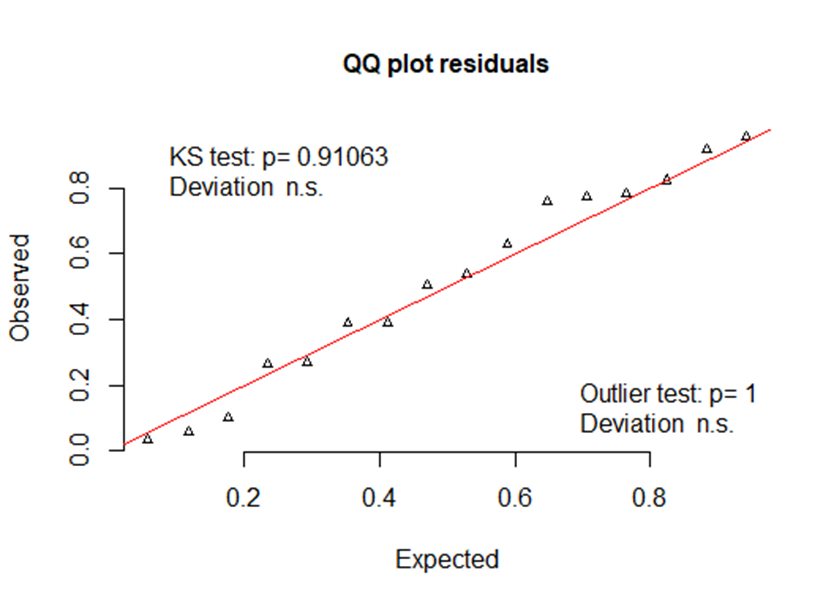
**

4**) Proportion of fights of females with males that were won by females versus proportion of males in the group**

Using N=16 group-year combination, a beta-binomial model was fitted to the proportion of female-male fights that were won by females. The fixed part of the model consisted of the proportion males in the group, and the random part consisted of crossed random effects of year and group.

The likelihood ratio test (LRT) of the full model versus the null model was significant (X^2^=16.3 on 3 d.f., P=-.001.

The pseudo R^2^ was 0.64 for the fixed and the random part together. For the fixed part alone the pseudo R^2^ was 0.0.62.

The residual based QQ-plot showed no evidence against the uniform distribution (KS test P=0.43; Outlier test P=1, see figure below).


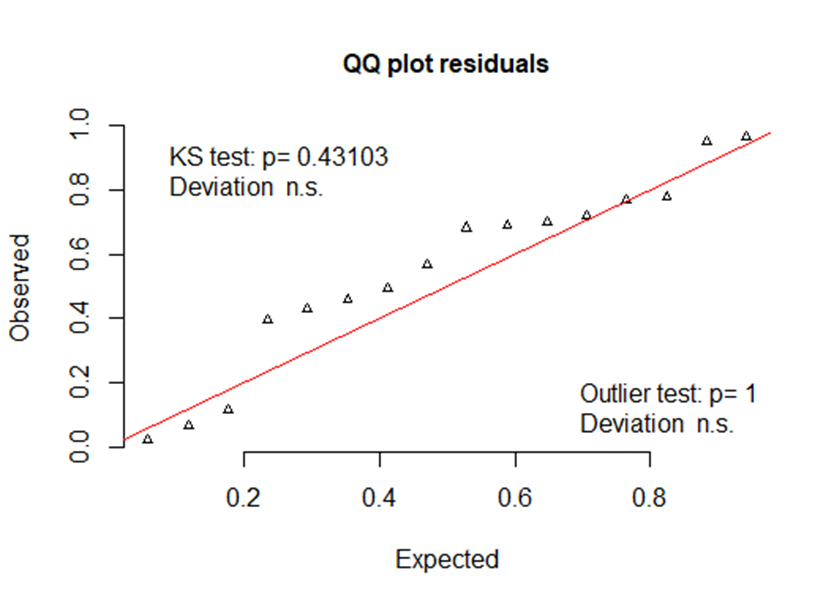


**5) Proportion of fights of females with males that females received support from males**

Using N=16 group-year combinations, a beta-binomial model was fitted to the proportion of of fights of females with males that females received support from males. The fixed part of the model consisted of the proportion of males in the group, and the random part consisted of crossed random effects of year and group.

The likelihood ratio test (LRT) of the full model versus the null model did not show a significant result (X^2^=1.48 on 3 d.f., P=0.69).

The pseudo R^2^ was 0.09for the fixed and the random part together. For the fixed part alone the pseudo R^2^ was 0.04.

The residual based QQ-plot showed no evidence against the uniform distribution (KS test P=0.73; Outlier test P=1, see figure below).


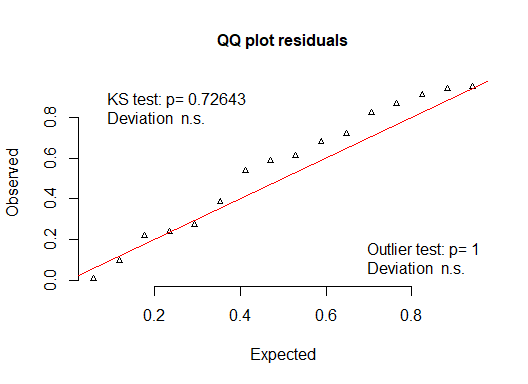


**6) Proportion of fights of females with males that females received support from females**

Using N=16 group-year combinations a beta-binomial model was fitted to the proportion of of fights of females with males that females received support from females. The fixed part of the model consisted of the proportion of males in the group, and the random part consisted of crossed random effects of year and group.

The likelihood ratio test (LRT) of the full model versus the null model was significant (X^2^=6.48 on 2 d.f., P=0.039; in this case we used the model without random group effect as full model, because its variance component was estimated as zero, leading to an undefined likelihood).

The pseudo R^2^ was 0.33for the fixed and random part together. For the fixed part alone the pseudo R^2^ was 0.29.

The residual based QQ-plot showed no evidence against the uniform distribution (KS test P=0.97; Outlier test P=1, see below).


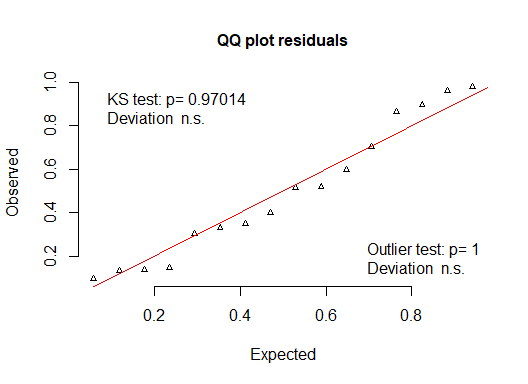


**7) Total aggression of males to females and its dependence on the mating season**

Using N=221 male-month combinations for 39 males, we fitted a truncated negative binomial model to the total frequency per month of aggressive acts per male to any of the females. The fixed part of the model consisted of the binary variable mating season (yes/no), and the random part consisted of crossed random effects of group and year, and nested random effects of group within year, and individual within group within year.

The likelihood ratio test (LRT) of the full model versus the null model was significant (X^2^=28.8 on 5 d.f., P<0.0001).

The pseudo R^2^ was 0.12 for the fixed and random part together. For the fixed part alone the pseudo R^2^ was 0.003.

The residual based QQ-plot showed no evidence against the uniform distribution (KS test P=0.15; Outlier test P=1, see below).


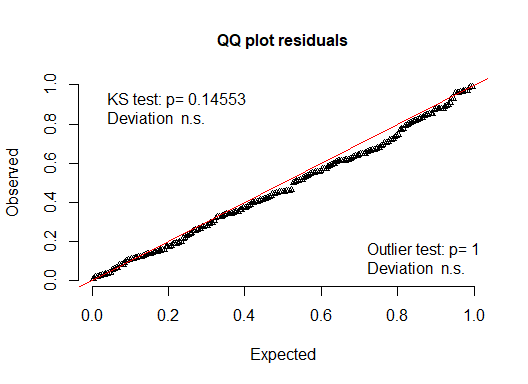


8) **Mild aggression of males to females and its dependence on the mating season**

Using N=194 male-month combinations for 38 males, we fitted a truncated negative binomial model to the frequency of acts of mild agression per individual male to any of the females per month. The fixed part of the model consisted of the binary variable mating season (yes/no), and the random part consisted of crossed random effects of group and year, and nested random effects of group within year, and individual within group within year.

The likelihood ratio test (LRT) of the full model versus the null model was significant (X^2^=14.1 on 5 d.f., P=0.015).

The pseudo R^2^ was 0.07 for fixed and random part together. For the fixed part alone the pseudo R^2^ was 0.01.


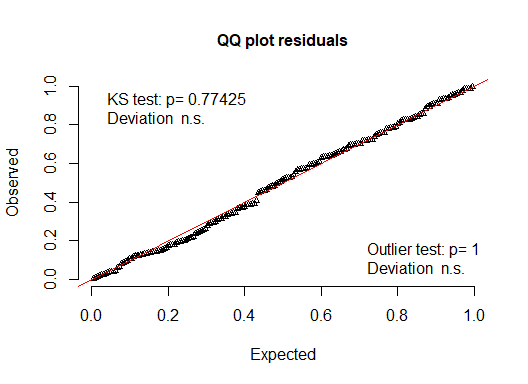
The residual based QQ-plot showed no evidence against the uniform distribution (KS test P=0.77; Outlier test P=1, see below).

9) **Severe aggression of males to females and its dependence on the mating season**

Using N=80 male-month combinations for 28 males, we fitted a truncated negative binomial model to the number of acts of severe aggression by an individual male to any of the females per month. The fixed part of the model consisted of the binary variable mating season (yes/no), and the random part consisted of crossed random effects of group and year, and nested random effects of group within year, and individual within group within year.

The likelihood ratio test (LRT) of the full model versus the null model was significant (X^2^=21.6 on 5 d.f., P=0.0006).

The pseudo R^2^ was 0.24 for the fixed and random part together. For the fixed part alone the pseudo R^2^ was 0.01.

The residual based QQ-plot showed no evidence against the uniform distribution (KS test P=0.15; Outlier test P=1, see below).


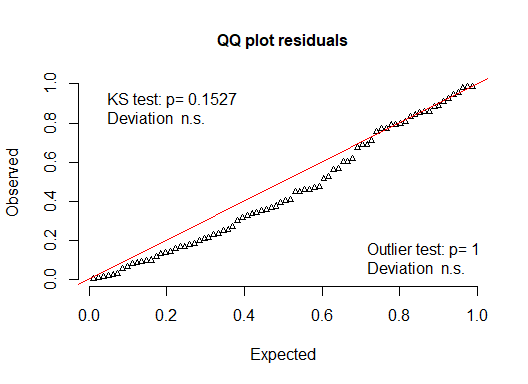


**II) Additional analyses** **based on correlations and combination methods**

In addition to using GLMMs, we also studied all patterns with correlations (Pearson and Kendall, where suited), Wilcox rank sum tests and Holm-Bonferroni methods, ignoring the repeated observations on some individuals that returned in different group-year points. This has led qualitatively to the same results, indicating the robustness of our results. We tested the data for normality using the Shapiro-Wilk test; if variables were normally distributed we used Pearson’s product-moment correlation tests, otherwise we used Kendall Rank correlation tests, both using the “cor.test” function in R. For the mating season analyses, we used Wilcox rank sum tests. To correct for multiple tests, we used the Holm-Bonferroni correction method and used separate alpha values for the tests examining our main hypothesis (4 comparisons) and the tests examining our alternative hypotheses (5 comparisons).

We showed that female dominance increased significantly with the proportion of males in a group (Pearson’s correlation, r = 0.61, t = 2.87, df = 14, p = 0.006, one-tailed, Holm-Bonferroni corrected alpha = 0.017).

We found that in groups with a higher proportion of males, males fight relatively more with other males as proportion of their interaction with both sexes (Kendall tau = 0.38, n=16, z = 2.04, p = 0.021, one-sided, Holm-Bonferroni corrected alpha = 0.05) and females win conflicts with males more often as a proportion of their winning conflicts with either sex (Kendall, tau = 0.46, z = 2.44, p-value = 0.007, Holm-Bonferroni corrected alpha = 0.025).

If we combine data of the three sites, female dominance over males increases significantly with proportion of males in the group (when using a Fisher combination test for the correlations in the three, separate sites, X^2^= 19.2, df = 6, p<0.005, one-sided, Holm-Bonferroni corrected alpha = 0.0125).

## Alternative hypotheses

## Coalitionary support

We found neither a correlation between proportion of males in the group and proportion of female-male conflicts in which the female was supported by another male (Kendall, tau = -0.16, z = -0.78, p = 0.78, n = 14, one-tailed, Holm-Bonferroni corrected alpha = 0.05, see Fig. 3a) nor by another female (Pearson, r = 0.21, t = 0.79, df = 14, p= 0.22, one-tailed, bonferroni corrected alpha = 0.025).

**Docile male hypothesis**

There was no significant effect of season on aggression by males towards females (mild aggression, W = 28, p = 0.048, Holm-Bonferroni corrected alpha = 0.01; fierce aggression, W = 24, p = 0.21, Holm-Bonferroni corrected alpha =0.017; total aggression; W = 27.5, p = 0.06, Holm-Bonferroni corrected alpha = 0.0125).
